# Meetings of Societies

**Published:** 1932

**Authors:** 


### Meetings of Societies

### Bristol Medico-Chirurgical Society

The fourth meeting of the session was held in the Physiological Lecture Theatre of the University, on Wednesday, 13th January, 1932, at 8.30 p.m., the President, Dr. J. R. CHARLES, in the Chair.

The minutes of the previous meeting were read and confirmed.

Dr. T. B. WANSBROUGH showed skiagrams and Dr. A. D. FRASER pathological specimens, including one of the **Brain from a Case of Carbon Monoxide Poisoning.** Dr. FRASER explained the pathology of this specimen, with the aid of the epidiascope. The PRESIDENT described the clinical history of this case and another similar one, and Dr. ALEXANDER and Dr. DIX also spoke on the case.

Dr. H. H. CARLETON then read a paper on "**Trigeminal Neuralgia,**" which appears on page 47 of this issue.

The PRESIDENT, in opening the discussion, stated that he had seen many cases successfully treated by drugs, and also some successes following avulsion. He regarded surgery as very drastic, and only to be used as a last resort. He suggested the use of diathermy in these cases.

Mr. G. F. FAWN described two cases successfully treated with diathermy after dental extraction had proved of no avail. He had also seen improvement following the use of ultra-violet rays, and suggested that the condition might sometimes be due to an ascending neuritis.

Mr. RENDLE SHORT congratulated Dr. Carleton on his paper, and agreed that injection was the method of choice. He used a general anæsthetic in these cases, using novocain first and watching the corneal reflex. He had not been so fortunate as Dr. Carleton in the matter of eye complications. He had found avulsion of the first division very good, and had no hesitation in advising operation if injection failed.

Mr. C. F. WALTERS said he had seen some depressing results from injection, including a case of cavernous sinus thrombosis. He would like to know the length of time over which these cases remained free from pain.

Mr. E. R. CHAMBERS emphasized the necessity of the care of the eye in these cases.

Colonel A. E. J. LISTER spoke of a case of hyperphoria following an injection.

Dr. A. T. TODD mentioned the value of trichlor-ethylene in these cases, and also of pyramidon combined with phenazone.

Mr. R. R. GARDEN spoke of the pathology of the keratitis which occurred in these cases, and suggested that all such eyes needed several months' treatment.

Mr. A. W. ADAMS wished to know the number of recurrences which had occurred in Dr. Carleton's series. He himself had seen six recurrences within a year in twenty-five cases. He used both the anterior and the lateral routes, and thought that a general anæsthetic was desirable. He had seen two damaged eyes, but thought that the risk was justifiable.

Dr. A. L. FLEMMING spoke of the technique in cases in which a general anæsthetic was used.

Professor E. W. HEY GROVES said that his own view was that treatment should start with drugs, followed by injection if these failed, with operation if injection proved of no value. He thought that the results of injection were often temporary, and had seen the greatest successes and also the greatest disasters following removal of the ganglion. He suggested injection following exposure of the ganglion by surgery.

Dr. W. P. KENNEDY said that these patients were only too glad to have any treatment, however dangerous. He thought that some mention should be made of gelsemium.

Dr. CARLETON in reply said that all his cases had been treated with drugs before they came to him, and suggested that many cures attributed to drugs were really natural remissions. He had no experience of diathermy, and had seen several failures with avulsion. He did not like general anæsthesia, and said that injection was not more painful than an attack. He said that recurrences were due to injection of the nerve instead of the ganglion, and that nearly all his cases were comfortable after three years. Three cases had asked for another injection.

---

The fifth meeting of the session was held in the Physiological Lecture Theatre of the University, on Wednesday, 10th February, 1932, at 8.30 p.m., Dr. J. R. CHARLES in the Chair.

The minutes of the previous meeting were read and confirmed.

Dr. A. L. TAYLOR showed a number of pathological specimens.

The PRESIDENT introduced to the Society Dr. C. S. MYERS, who gave an address on "**Human Improvability,**" published on page 31.

Following the address questions were asked by the President, Mr. Walters, Dr. Rudolf, Dr. Frank Bodman, and Dr. Carleton.

Dr. MYERS replied.

Professor HEY GROVES proposed a vote of thanks to Dr. Myers for his address ; this was seconded by Dr. R. G. GORDON and carried with acclamation.

Dr. MYERS thanked the Society.

---

The sixth meeting of the session was held in the Physiological Lecture Theatre of the University, on Wednesday, 9th March, 1932, at 8.30 p.m. Dr. J. R. CHARLES in the Chair.

Dr. BERGIN and Dr. A. D. FRASER showed respectively X-Rays and specimens.

Dr. G. E. F. SUTTON demonstrated a case of **Congenital Pseudo-hypertrophic Muscular Paralysis.**

Dr. A. T. TODD then read a paper on "**The Medical Treatment of Graves's Disease and Toxic Goitre,**" and showed several cases to illustrate the effect of his treatment.

The PRESIDENT, in opening the discussion, said that Graves's disease was a condition whcih had been successfully treated by many different branches of the profession. He had had two cases which had doubled their weight under medical treatment. He agreed that diet and rest were important, but also used codeia. He never gave iodine, and wondered if X-ray treatment of the adrenals might not prove dangerous from over-dosage.

Mr. RENDLE SHORT paid a tribute to Dr. Todd for the courage and originality of his work, but said that, in his opinion, surgery still remained the best treatment for cases of over six months' standing. He spoke of his own findings in Dr. Todd's cases, and said that they did not all come up to his standard of clinical cure. He had operated on well over one hundred cases with a mortality of 8 per cent.

Dr. H. H. CARLETON spoke of the value of surgery in this condition, and emphasized the importance of gentle surgery.

Dr. J. O. SYMES said that this was a serious disease, and quoted figures from his own experience of thirty cases with a 20 per cent. mortality, and none of those living able to do a day's work. He said that hydrofluoric acid had been a favourite treatment in his younger days.

Dr. C. E. K. HERAPATH would have liked more details of Dr. Todd's cases, and spoke of the value of surgery in hastening the cure. At the present time he was using radium and spoke well of the results.

Mr. S. J. H. GRIFFITHS was doubtful if Dr. Todd had proved his case, and said that the criterion of cure must be the patients' ability to return to their own work. He quoted figures from the Mayo Clinic and from his own series of ninety-four surgical cases with three deaths.

Dr. R. H. BURNETT suggested the possibility of giving sodium fluoride as a palatinoid.

Dr. FRANK BODMAN wished to know more of the biochemistry of these conditions, particularly with regard to the chloride content of the blood.

Dr. NEWMAN NEILD congratulated Dr. Todd, and hoped that he had started a return of these cases to the physician, similar to that which had occurred in the treatment of gastric ulcer. He spoke of the value of the instillation of a drop of castor oil in the conjunctival sac in cases of exophthalmos.

Dr. G. E. F. SUTTON spoke of the value of codeia as used by the President, and of the use of the basal metabolic rate in diagnosis and control.

Dr. D. A. ALEXANDER mentioned the increase in the number of cases in recent years, and spoke of the importance of the psychological attitude of the medical attendant.

Dr. R. C. CLARKE spoke of the difficulty and importance of exact diagnosis in these cases, and said that successes had been claimed for many peculiar treatments in the past.

Dr. TODD, in reply, said that this was not a "stunt" treatment. The cases he had shown were all recent cases under treatment, and that they had undergone a very severe test in being shown to a crowded meeting. There were forty-four other cases, nearly all of whom were back at their work. He did not think there was any danger of Addison's disease arising as a result of X-rays to the adrenals. He was very interested to hear of the hydrofluoric acid treatment, but pointed out the difficulties in dispensing this drug. He thought there was little value in the basal metabolic rate, and did not use it now. He considered that his treatment was perfectly safe.

The PRESIDENT summed up the discussion in a few words.

